# Bibliographical

**Published:** 1879-10

**Authors:** 


### BIBLIOGRAPHICAL.

### THE ORIGIN AND FORMATION OF THE DENTAL FOLLICLE.

*By Drs. Legros and Magitot.*

This work has just been translated from the French by Dr. M. S. Dean, of Chicago.

It is an exhaustive treatise upon this subject. The tissues from which the teeth originate and the progressive stages of development, are minutely described and amply illustrated, from their incipiency to the completion of the dental follicle. This covers ground that had been only partly explored by previous investigators. These authors have here undertaken the most difficult task that the embryologist has to deal with in connection with the development of the teeth. In addition to the human embryo, their investigations have been made upon large numbers of those of the different species of domestic animals.

The authors commence their description with the elements that constitute the external stratum of the mucous membrane, and at an epoch anterior to the full development of the subjacent tissues: thence tracing their history, step by step, in the formation of the enamel organ, down to the period at which the dentine-papilla makes its appearance. They then describe the progressive stages of these and the surrounding tissues, both separately and as a whole. In this way they give to the reader a very complete idea of the character and mode of development of the histological elements that enter into the constitution of the teeth. In all the morphological changes that take place in these cells, and the different physiological functions they are called upon to perform, the primitive origin and character of these elements are always kept prominently in view. The physiology of these parts is considered in connection with their histology, and with the same thoroughness of research and minuteness of detail. While many authors have described the process of development of the hard tissues

—the enamel and dentine—the stages of evolution prior to the formation of these have been left in comparative obscurity; the origin and development of the enamel organ, the dentine-papilla and the adjacent parts, having been passed over lightly, and without giving a definite idea of their embryonal phenomena. From this treatise, therefore, we get a clearer insight into these occult processes of evolution than from any other work.

The translator's introduction occupies about forty pages and treats of the Mucous Membrane, The Growth and Decline of Meckel's Cartilage, the Intermaxillary Bones, etc.

The description of the mucous membrane and the terminology used, are very much simplified; and, with the aid of the diagrams and cuts, the elements from which the enamel organs originate may be easily understood, The writer gives a clearer and more intelligible idea of the position of the layers of the mucous membrane and their elements, especially those from which the enamel and dentine are derived than has hitherto been done. This description was not necessary for such as are familiar with the minute anatomy of these parts, as those were supposed to be for whom the work was originally written; to many, however, who have not gone over the ground thoroughly, it will be found a valuable aid in clearly comprehending the subsequent treatise.

The article on Meckel's Cartilage is exceedingly interesting and instructive. A description of this organ is given, from its first appearance as a temporary support to the lower jaw, till it finally disappears. This account of a structure, very little understood in our country, is made additionally clear by cuts drawn from nature by Magitot and Robin, and the translator. It gives a more definite idea of this organ, than is elsewhere found in the English language.

The original treatise is amplified by the translator's explanatory notes, in which the views of many of the more eminent authors have been introduced; generally favoring, but sometimes dissenting, from the views advanced in the text. From these, the reader will gain considerable knowledge of the recent literature upon many difficult and disputed points in the evolution of the teeth, which he would otherwise find it difficult to obtain. This we consider an important feature of the book.

"Although this work may not be regarded as a practical treatise in the ordinary acceptation of the term, yet it contains valuable lessons, which may be applied in remedying the disasterous consequences of those exanthematous diseases, which are so liable to arrest or pervert the nutritive supply, and which do often result in the disfigurement of these developing organs, and sometimes in their loss. Hence its suggestions should not be confined to the dental surgeon alone, but should be made available by the medical profession who, at the present day, retain almost undisputed possession of this sadly neglected field of practice."

The book is illustrated not only from the very superior cuts, copied from the original, (which represent to the eye, the various stages of development more fully than any preceding work;) but also by several that have been added by the translator, of which some are drawn from nature, and some are selected from the works of recent authors.

The translator has achieved the difficult task of giving the exact sense of the authors, so far as we have been able to discover, and at the same time of presenting their ideas in language entirely free from the harsh and inelegant style, so common in translations of scientific works. For this, and for the entire undertaking, he is entitled to the thanks of all who desire to see the profession of dentistry resting upon a scientific foundation, and legitimately entitled to rank among the learned professions; and if the work is properly appreciated, it will soon be found in the hands of every dental practitioner and student in the land.

